# Using Natural Language Processing to Explore Patient Perspectives on AI Avatars in Support Materials for Patients With Breast Cancer: Survey Study

**DOI:** 10.2196/70971

**Published:** 2025-06-20

**Authors:** Eleanor Cheese, Raouef Ahmed Bichoo, Kartikae Grover, Dorin Dumitru, Alexandros Zenonos, Joanne Groark, Douglas Gibson, Rebecca Pope

**Affiliations:** 1 Roche Products Ltd UK Welwyn Garden City United Kingdom; 2 Hull University Teaching Hospital NHS Trust Hull United Kingdom

**Keywords:** educational videos, breast cancer, natural language processing, avatars, patient feedback, artificial intelligence, AI

## Abstract

**Background:**

Having well-informed patients is crucial to enhancing patient satisfaction, quality of life, and health outcomes, which in turn optimizes health care use. Traditional methods of delivering information, such as booklets and leaflets, are often ineffective and can overwhelm patients. Educational videos represent a promising alternative; however, their production typically requires significant time and financial resources. Video production using generative artificial intelligence (AI) technology may provide a solution to this problem.

**Objective:**

This study aimed to use natural language processing (NLP) to understand free-text patient feedback on 1 of 7 AI-generated patient educational videos created in collaboration with Roche UK and the Hull University Teaching Hospitals NHS Trust breast cancer team, titled “Breast Cancer Follow Up Programme.”

**Methods:**

A survey was sent to 400 patients who had completed the breast cancer treatment pathway, and 98 (24.5%) free-text responses were received for the question “Any comments or suggestions to improve its [the video’s] contents?” We applied and evaluated different NLP machine learning techniques to draw insights from these unstructured data, namely sentiment analysis, topic modeling, summarization, and term frequency–inverse document frequency word clouds.

**Results:**

Sentiment analysis showed that 81% (79/98) of the responses were positive or neutral, while negative comments were predominantly related to the AI avatar. Topic modeling using BERTopic with k-means clustering was found to be the most effective model and identified 4 key topics: the breast cancer treatment pathway, video content, the digital avatar or narrator, and short responses with little or no content. The term frequency–inverse document frequency word clouds indicated positive sentiment about the treatment pathway (eg, “reassured” and “faultless”) and video content (eg, “informative” and “clear”), whereas the AI avatar was often described negatively (eg, “impersonal”). Summarization using the text-to-text transfer transformer model effectively created summaries of the responses by topic.

**Conclusions:**

This study demonstrates the success of NLP techniques in efficiently generating insights into patient feedback related to generative AI educational content. Combining NLP methods resulted in clear visuals and insights, enhancing the understanding of patient feedback. Analysis of free-text responses provided clinicians at Hull University Teaching Hospitals NHS Trust with deeper insights than those obtained from quantitative Likert scale responses alone. Importantly, the results validate the use of generative AI in creating patient educational videos, highlighting its potential to address the challenges of costly video production and the limitations of traditional, often overwhelming educational leaflets. Despite the positive overall feedback, negative comments focused on the technical aspects of the AI avatar, indicating areas for improvement. We advocate that patients who receive AI avatar explanations are counseled that this technology is intended to supplement, not replace, human health care interactions. Future investigations are needed to confirm the ongoing effectiveness of these educational tools.

## Introduction

### Background

Efforts to ensure that patients are well informed can improve patient satisfaction, quality of life, and health outcomes, which in turn optimizes health care use [1]. However, traditional passive methods of delivering information to patients through booklets and leaflets can prove ineffective and overwhelm patients [2]. Moreover, printed materials are fixed in their language delivery: in English-speaking health care settings, for example, they are typically produced in English, which may exclude patients who do not speak English or have visual impairment. Similarly, a person with good conversational fluency in English may not be able to understand, discuss, or read health-related information proficiently in English [3], which can worsen health inequalities and outcomes [4]. In England, this issue is further exacerbated by the ongoing workforce crisis in the National Health Service (NHS) [5], leaving patients with breast cancer on treatment pathways struggling to access accurate and timely information.

To address these challenges, innovative solutions are necessary to streamline health care professionals’ workloads, enabling them to focus their specialist skill set on patient care. One promising approach involves the use of patient educational videos. More specifically, with the rise of generative artificial intelligence (GenAI), it is now possible to quickly create these educational videos at low cost, using digital avatars to narrate tailored scripts and incorporating language preferences that meet the specific information needs of viewers.

Hull University Teaching Hospitals NHS Trust (HUTH) experienced such pressures within their breast cancer service. Specialist breast cancer nurses were spending significant amounts of time on the telephone answering common patient queries on general topics (eg, “What happens at the end of my cancer treatment?” “What are the signs of recurrence that I need to look out for?” “Who do I contact if I have concerns?”), which diverted time away from addressing highly specialized, treatment-related questions. Being aware of the benefits of educational videos in mitigating this issue and in supporting shared decision-making in consultations, HUTH explored the process of making videos manually. However, this proved to be time consuming and expensive, and it was difficult to produce videos to professional standards. Recognizing this issue, Roche UK and HUTH identified an opportunity to pilot educational videos that leveraged GenAI to create digital avatars [6,7], reducing the time and financial resources required to produce educational videos.

The primary aim of this pilot was to streamline the breast cancer treatment pathway and collect feedback from patients on whether they felt that these digital avatars improved their experience. Seven educational videos were developed in collaboration with clinicians and breast surgeons, ensuring that the content was precisely tailored to the informational needs of patients with breast cancer at various stages of their health journey [8]. The videos’ contents covered a range of topics, including the “Breast Cancer Follow Up Programme,” which outlined a patient’s journey after their cancer treatment.

To assess the success of this initiative, a survey was sent via email to 400 HUTH patients who were in remission and who had participated in watching the “Breast Cancer Follow Up Programme” retrospectively. The survey included quantitative Likert scale questions as well as free-text open-ended questions. Analyzing free-text responses offers significant advantages over relying solely on insights from Likert scale responses because free-text responses allow patients to describe their experiences in their own words, providing deeper and more specific insights and context that closed-ended questions may miss. Furthermore, free-text responses can enable the identification of emerging themes in patient perspective, such as areas of concern, suggestions for improvement, or positive aspects [9-11]. Therefore, the analysis of free-text responses is essential for understanding the nuances of patient experiences and for revealing actionable insights. However, free-text responses are often difficult to analyze in large quantities, especially in clinical settings, because clinical time is constrained, and it is often impractical for clinical staff to manually review survey feedback. Therefore, natural language processing (NLP) techniques were used to help draw insights and capture patient experiences from these data.

The aim of this work was to analyze qualitative survey feedback received in free-text (unstructured) format for the video “Breast Cancer Follow Up Programme” [8]. This video was selected due to the large number of patients who had experienced the breast cancer service.

### Prior Work

Recent advancements in technology, particularly in AI and GenAI, have influenced efforts to improve patient care [12-14]. GenAI has predominantly been applied in health care in areas such as screening and diagnosis, clinical administration support, decision support, professional medical education, and patient engagement chatbots [15,16]. However, few studies have explored the use of GenAI to create patient educational videos or the subsequent application of traditional artificial intelligence (AI) methods, such as NLP, to understand patient preferences regarding this method of delivering patient education.

Some health care providers have implemented GenAI to develop multilingual videos aimed at both health care professionals and patients [17,18]. Notably, Adeboye et al [19] explored the use of avatar-narrated videos to educate patients in postoperative wound care after breast surgery, with survey results indicating that 79% of the patients preferred this mode of content delivery over traditional leaflets. To our knowledge, our study is the first to use NLP techniques to analyze patient feedback on the use of GenAI videos.

Patient centeredness is essential for high-quality care [20-22], leading to a focus on integrating patient experiences and feedback into care delivery [23,24]. Patient experience surveys often include closed-ended questions for quantitative insights and open-ended questions for richer, detailed feedback [25]. While closed-ended questions are easier to analyze [26], open-ended responses are valued higher by health care professionals for identifying important topics and providing context to the closed-ended questions [9,27]. Gallan et al [28] noted that survey comments can sometimes contradict qualitative results, emphasizing the need to consider both to fully understand patient experiences.

Qualitative patient responses, although valuable, are often underused in clinical practice due to the effort required for manual analysis [10,29]. Data science techniques, particularly NLP, offer a solution by enabling efficient analysis of free-text responses [30]; for instance, many studies use topic analysis, an NLP method that structures large text datasets into topics [28,31-35]. Common approaches include topic modeling, an unsupervised method in which algorithms identify topics; and topic classification, a supervised method in which texts are assigned to predefined topics. There are benefits and disadvantages to both approaches depending on the use case; however, in terms of topic quality, 2 studies have concluded similar results from both approaches [36,37].

Cammel et al [38] applied unsupervised topic modeling to free-text patient experience data from 2 hospitals. Using nonnegative matrix factorization (NMF), the authors selected a model with the highest topic coherence, assigning 87% of the responses to topics. They noted the model’s transferability to data from other hospitals due to its unsupervised nature.

Additional benefits of unsupervised models over supervised classification include reduced human bias because predefined categories are unnecessary. Conversely, Doing-Harris et al [36] used a supervised approach, applying vocabulary-based and naive Bayes classifiers with 28 predefined topics adapted from the taxonomy of patient satisfaction themes proposed by López et al [39], with naive Bayes performing better. The authors also applied topic modeling using latent Dirichlet allocation (LDA) to negative comments, which revealed no new topics, suggesting that accurate predefined topics can eliminate the need for topic discovery with unsupervised models.

Sentiment analysis is another NLP technique commonly used in the analysis of patient survey responses [28,31,40], wherein a piece of text is assigned a score or likelihood indicating its sentiment as negative, neutral, or positive. The choice of a pretrained model is crucial because its decisions depend heavily on the training data. Cammel et al [38] used the *pattern.nl* package (trained on product reviews) to categorize their patient data but faced issues related to domain specificity; for instance, “disease” was classified as negative when, in this context, it should have been classified as neutral. By contrast, van Buchem et al [29] fine-tuned a pretrained bidirectional encoder representations from transformers (BERT) model with manually labeled data, achieving up to 97% accuracy.

Combining NLP techniques can provide deeper insights; for example, Cammel et al [38] combined topic analysis and frequency to create a matrix that categorized topics into “topics to improve” (frequently mentioned topics with negative sentiment), “topics to celebrate” (frequently mentioned topics with positive sentiment), and “topics to monitor” (frequently mentioned topics with neutral sentiment and infrequently mentioned topics with negative sentiment). Word clouds are another way of visualizing commonly occurring words and themes within patient responses. Khanbhai et al [9] combined word clouds with topic analysis to reveal ideas by health care setting (eg, outpatient vs inpatient), whereas Nawab et al [41] combined word clouds with sentiment analysis to show common complaints and compliments, with separate word clouds for negative and positive comments.

Summarization, a subfield of NLP, has been a subject of study for several decades but has advanced rapidly and significantly in recent years, primarily due to the development of large language models (LLMs). Within the health care domain, LLM summarization techniques have predominantly been explored in the context of electronic health records and clinical notes summarization [42,43]; for example, Tariq et al [44] used the text-to-text transfer transformer (T5) model to generate layperson-friendly summaries of clinical radiology notes, while Van Veen et al [45] evaluated various LLMs across a range of clinical text summarization tasks and found that fine-tuned language net–T5 (FLAN-T5) and GPT-4 performed best overall.

### Objectives

This work built on previous efforts by testing unsupervised topic models not previously applied to patient data and combining word clouds in a novel way with sentiment and topic analysis to create sentiment-based color-coded word clouds per topic. In addition, we used LLM summarization to summarize patient experience data by topic, an innovative application in this context.

The aims of this study were as follows:

1. To investigate the utility of AI-generated patient educational videos to deliver information on the breast cancer treatment pathway from the patient perspective
2. To understand whether NLP techniques can generate automated insights into qualitative patient feedback

## Methods

### Data

The raw patient data survey collated by HUTH included patient-identifiable data (eg, email addresses, age brackets, and hospital numbers), Likert scale responses, and free-text answers to the question “Any comments or suggestions to improve its [the video’s] contents?” For the purposes of this research and to adhere to the contractual data-processing and data-sharing agreement between Roche UK and HUTH, all patient-identifiable information was removed before data transfer to Roche UK.

From the 400 patient surveys sent electronically, 255 (63.8%) responses were collected. Of the 255 respondents, 98 (38.4%) answered the open-ended question. NLP analysis was conducted on these free-text responses. All survey questions—closed ended as well as open ended—were optional. The 12 closed-ended questions had response rates ranging from 98.8% (252/255) to 100% (255/255), with 9 (75%) achieving a 100% (255/255) response rate.

**Figure 1:**
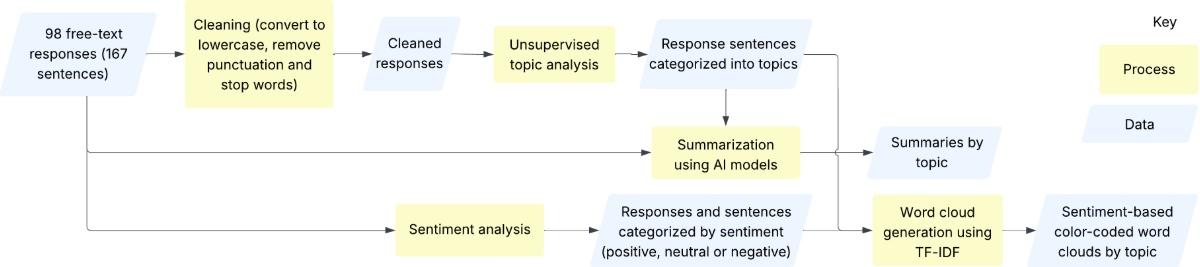
Flowchart overview of the survey data analysis process, with examples of generated visualizations. AI: artificial intelligence; TF-IDF: term frequency–inverse document frequency.

As depicted in Figure 1, data cleaning was applied to the raw survey responses to support NLP techniques used in this study (specifically topic analysis and the generation of word clouds using term frequency–inverse document frequency [TF-IDF]). These techniques aim to reduce the dimensionality (ie, the number of words used) of the responses, and data cleaning facilitates this process. The responses were cleaned by expanding contractions, lemmatizing each word, and removing stop words (noninformative words such as “the” and “of”) and punctuation (except for full stops, which were retained to enable sentence-level analysis). For sentiment analysis and summarization, the raw data were used because punctuation and stop words, including negation or intensifier words, can impact the sentiment of a sentence and affect the overall meaning. To allow for deeper analysis, responses were split into individual sentences (n=167) because it is possible that patients wrote about multiple topics and expressed varying sentiments within a single response. Data cleaning was achieved using the *Natural Language Toolkit* (*NLTK*) Python library [46].

### Analysis Pipeline

NLP analysis methods were applied to both raw and cleaned data to generate meaningful insights from the unstructured patient feedback data, specifically topic and sentiment categorization of full responses and response sentiments, summaries by topic, and sentiment-based color-coded word clouds as illustrated in Figure 1 and described in detail in the next subsection.

### NLP Methods

All analyses were conducted using Python (version 3.9.12; Python Software Foundation).

#### Sentiment Analysis

To determine the sentiment of each of the patient responses, Valence Aware Dictionary and Sentiment Reasoner (VADER) was applied to the response sentences using the *VADER* Python package [47]. VADER is a rule-based sentiment analysis tool that relies on sentiment dictionaries, in which each word has an associated sentiment score, to determine the overall sentiment of a text. The model is pretrained on social media comments, making it well suited for predicting the sentiment of short, informal texts containing expressed opinions, similar to the response data in this study. However, a manual inspection of the model’s predictions revealed that it did not reliably classify sentiment. As noted previously, this is likely due to words that are typically negative in general context (eg, “disease” and “cancer”) but should be classified as neutral, given the context of the survey.

Therefore, a pretrained machine learning model was used instead, namely the popular HuggingFace model Twitter-roBERTa-base for Sentiment Analysis - Updated [48], which was built using the Robustly Optimized BERT Pretraining Approach (RoBERTa) transformer architecture and trained on 124 million tweets. This model was chosen because the type of data it was trained on (tweets) is similar to that analyzed in this project (patient feedback): both contain opinions and emotional language, occasionally accompanied by punctuation or emojis to express sentiment. The model is able to process this emotional language, taking into account punctuation and emojis, both serving as important indicators of sentiment intensity used to convey a person’s emotional state. A manual inspection showed that the model reliably predicted the sentiment of the responses. Therefore, it was used to categorize both full responses and individual sentences as having positive, neutral, or negative sentiment, based on the highest probability score.

#### Topic Analysis

Given that the survey was based around feedback for the patient education video, the themes in our data were unlikely to reflect those commonly found in most patient satisfaction surveys, which tend to center on hospital or care experiences. Therefore, instead of classification approaches, we used unsupervised topic modeling techniques to allow for the identification of unexpected topics. Three popular unsupervised topic models—LDA, NMF, and BERTopic—were applied to the cleaned data, using the *Gensim* Python library [49] for LDA and NMF and the *BERTopic* Python library for BERTopic [50]. Each model was tested with a varying number of topics (ranging from 2 to 10) and evaluated manually for the meaningfulness and contextual relevance of the identified topics as well as topic coherence (Table 1). Topic coherence refers to the degree of semantic similarity among the topic descriptors [51].

**Table 1:** Highest topic coherence scores for the unsupervised topic models applied to cleaned patient response sentences, each tested with a varying number of topics (ranging from 2 to 10).

Models	Topic coherence score (rounded to 4 significant figures)
LDA^a^	0.4844
NMF^b^	0.4845
BERTopic (cHDBSCAN: hierarchical density-based spatial clustering of applications with noise)	0.5607
BERTopic (KeyBERT)	0.5514
BERTopic (k-means clustering with 4 clusters)	0.5467
BERTopic (KeyBERT and k-means clustering with 4 clusters)	0.5630

^a^LDA: latent Dirichlet allocation.

^b^NMF: nonnegative matrix factorization.

LDA and NMF are traditional algorithms widely used for topic modeling. However, these methods offer little interpretability or insight and yielded the lowest topic coherence scores. BERTopic [49], a more advanced model, was also tested using various hyperparameters. On the basis of topic coherence scores and a manual inspection of the topics and constituent words, BERTopic was the most effective at identifying meaningful topics. Once the optimal number of topics (4) was identified using the elbow method (by plotting the coherence score against the number of topics), BERTopic was reapplied with a k-means clustering model, with the number of clusters set to 4. This allowed for a visual inspection of the clusters and ensured that each sentence was assigned to a topic. This method, when combined with KeyBERT (a keyword extraction technique, implemented using the *KeyBERT* Python package [52]), achieved the highest topic coherence score (Table 1). The identified topics were visualized by reducing the dimensionality of the data using Uniform Manifold Approximation and Projection, implemented via the *Uniform Manifold Approximation and Projection-learn* Python library [53,54], and plotting the clusters on a 2D scatter plot.

#### Word Clouds

A word cloud was created for each topic identified through topic analysis to display the keywords used within the response sentences belonging to that topic. Keywords were identified using TF-IDF [50], implemented with the TFidfVectorizer from the *scikit-learn* Python library [55] using the following formula:

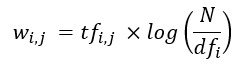



where *tf_i,j_* is the frequency of *i* (the word) in *j* (sentences belonging to the topic in question), and *df_i_* is the number of documents (sentences) containing *i* across all sentences.

Therefore, the size of each keyword in the word cloud corresponds to how frequently the word appeared in response sentences within a given topic, taking into account how often it appeared across all topics. Keywords were color coded by sentiment: red for negative, orange for neutral, and green for positive. For words appearing in multiple sentences, sentiment probabilities were summed, and the sentiment with the highest probability determined the overall sentiment context for that word. Word clouds were generated using the *wordcloud* Python package [56].

#### Summarization

There are 2 main methods of text summarization: extractive summarization, which involves selecting important sentences from the input text; and abstractive summarization, which involves generating new, paraphrased content that captures the main ideas from the input text. In this study, abstractive techniques were used to produce condensed, coherent summaries, while also ensuring that no patient response data were revealed. LLMs are powerful and popular tools for performing abstractive summarization because they are pretrained on massive datasets, enabling them to learn complex language patterns and semantics. Popular LLMs include GPT-4 (OpenAI) and Claude (Anthropic). However, because these tools are accessible only through application programming interfaces, their use would have compromised the confidentiality of the patient data [57]. Therefore, to summarize the responses from each of the 3 informative topics identified through topic analysis (excluding the topic comprising short responses with little or no content), 5 popular open-source downloadable abstractive text summarization models—Pegasus [58], GPT-2 [59], bidirectional and auto-regressive transformers [60], T5 [61], and the newer FLAN-T5 model [62]—were tested locally, using the *transformers* Python library [63]. These models were chosen due to their popularity and well-established abilities to perform zero-shot abstractive summarization at a high standard [58-62]. As human-written ground truth or “gold” summaries were not available due to time constraints and lack of resources, preference-based manual evaluation was used to compare the summaries generated by the various models. Upon reviewing the summaries against the raw data, T5 was found to be the best model for summarizing the feedback because its topic summaries best covered the main ideas pertinent to each topic without revealing raw patient response data. These T5-generated summaries were then manually edited slightly for grammatical clarity.

Summaries were also generated for responses separated by topic and sentiment using each of the models; however, this level of stratification resulted in few responses per group to summarize. Consequently, the outputs of the summarization models were of poor quality, often generating nonsensical results such as repeated short phrases from the responses. Therefore, only summaries generated at the topic level were used.

### Ethical Considerations

This collaborative project between Roche UK and HUTH was approved by the HUTH legal team and chief medical officer. Regarding the collection of patient feedback via electronic surveys, Trust policy requires all audits to be registered with the trust governance system; however patient surveys are exempt [64,65]. Therefore, ethical review and approval from the NHS research ethics committee were not required. Patients were given the choice to participate in this study and respond to the survey, and only those who consented provided responses. Response data were anonymized before being provided to Roche UK by removing patient email addresses. No compensation was provided to participants.

## Results

### Sentiment Analysis

Almost half of the patient responses (48/98, 49%) were positive, a little less than one-third (31/98, 32%) were neutral, and slightly less than one-fifth (19/98, 19%) were negative (Table 2). Responses were also split into constituent sentences and categorized by sentiment using the same NLP sentiment categorization method applied to the full responses. Analysis of the individual sentences revealed that the proportion of positive response sentences decreased slightly, whereas the proportion of neutral and negative response sentences increased. This indicates that some patient responses classified as overall positive likely contained a mixture of both positive and negative sentiment.

**Table 2:** Sentiment analysis results for each patient response and each sentence in the responses.

Sentiment	Responses (n=98), n (%)	Sentences (n=167), n (%)
Positive	48 (49)	72 (43.1)
Neutral	31 (31.6)	56 (33.5)
Negative	19 (19.4)	39 (23.4)

### Sentiment Distribution by Rating

**Figure 2:**
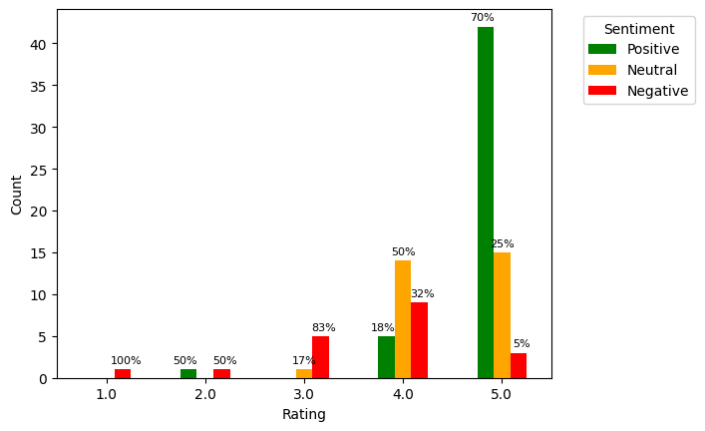
Grouped bar graph showing free-text response sentiment distribution by video rating (response to the question “Your overall rating of this video [score between 1-5 with 5 = excellent and 1 = very poor]”).

**Figure 3:**
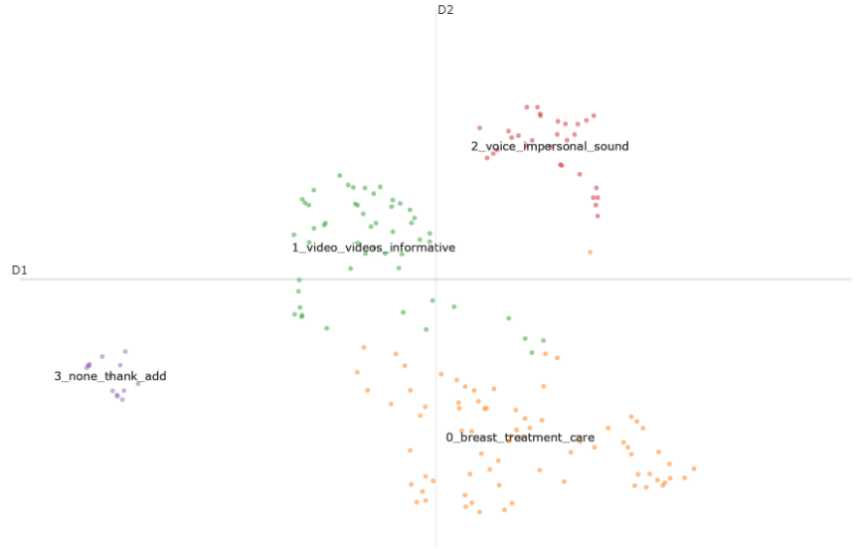
A visualization of the topic clustering analysis of free-text response sentences. Each data point represents a patient response, color coded and labeled by the topic to which it has been assigned. Topic labels consist of the 3 most relevant words within sentences of that topic (as identified by term frequency–inverse document frequency). The distance between data points represents semantic similarity (greater distance indicates less similarity).

**Figure 4:**
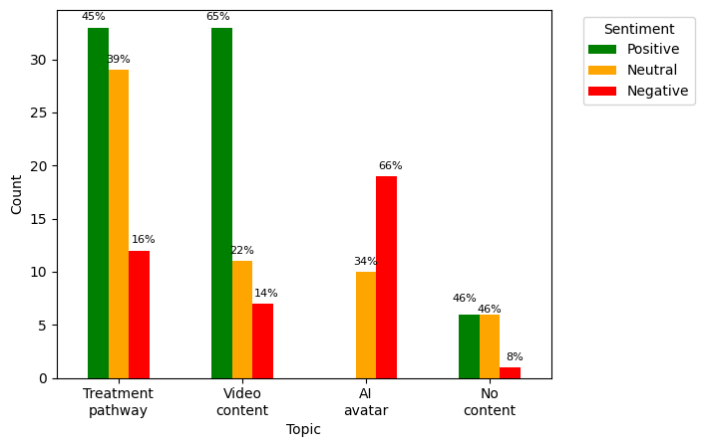
Grouped bar graph showing free-text response sentiment distribution of sentences assigned to each topic. AI: artificial intelligence.

To assess whether patients’ response sentiment correlated with their overall rating of the video, a grouped bar graph was plotted. Figure 2 shows that most of the patients (42/60, 70%) who rated the video with the maximum score (5) expressed positive sentiment in their free-text responses. As the rating decreased, the proportion of positive responses decreased, while the proportion of negative responses increased. This pattern is logical because the patients’ overall satisfaction or dissatisfaction with the video would be reflected consistently throughout their survey responses. Furthermore, this helped validate that the sentiment analysis method used was accurate. A manual inspection was conducted of any unusual results, such as negative responses from patients who rated the video as excellent (score of 5), and verified that these were indeed correctly categorized because their content was overall negative. Of the 3 such responses, 1 (33%) included a comment about disliking the avatar wearing black, which can be taken as a suggestion for improvement. The other 2 negative responses were not about the video; rather, 1 (50%) was regarding the survey itself (the respondent found it confusing); and 1 (50%) described the respondent’s negative experience of feeling unsupported during their treatment, although the respondent expressed confidence that the video would help others feel supported. This highlights a limitation of analyzing the sentiment of the whole response as an indicator of respondents’ sentiment regarding the video because the response may not discuss only the topic of the video. Similarly, neutral responses with a video rating of 5 tended to be those that did not contain informative content or discuss the videos (eg, “No comments” or “None”). Analysis at the topic and sentence levels (Figure 3 and Figure 4, respectively) provided deeper insight into patients’ perspectives on the videos.

### Topic Analysis

Four topics were identified by the BERTopic model (Table 3).

K-means clustering was used to assign each sentence to a topic—ensuring that no responses were excluded from the analysis—and to visually inspect the topics. As shown in Figure 3, topic 0 (treatment pathway) and topic 1 (video content) are fairly overlapping topics, with some patient responses falling somewhere in between the 2 topics. Other topics are far apart, indicating minimal semantic similarity.

**Table 3:** Topics identified by BERTopic with k-means clustering and KeyBERT.

Topic representative words	Topic name
0: breast_treatment_care	Treatment pathway^a^
1: video_videos_informative	Video content^b^
2: voice_impersonal_sound	AI^c^ avatar^d^
3: none_thank_add	No content^e^

^a^Topic 0: these responses tended to be about the treatment pathway and how the video fit into it or the patients’ experience of how the staff or literature supported them.

^b^Topic 1: these responses concerned the video content and the patients’ opinions on the information provided.

^c^AI: artificial intelligence.

^d^Topic 2: these responses concerned the AI presenter, its voice, and a few other technical aspects of the video, such as the subtitles or the audio.

^e^Topic 3: these responses were very short, such as “No comments” or “Thank you,” which did not provide any insight.

### Topic Popularity

To assess the popularity of each topic in patients’ feedback, the number of patients who mentioned each topic in their responses was calculated. As shown in Table 4, the treatment pathway topic was the most popular, with half of the patients (49/98, 50%) who gave free-text feedback mentioning this topic. The next most popular topic was video content, with a little less than half of the patients (43/98, 44%) mentioning this topic. Only 17% (17/98) of the patients mentioned the AI avatar.

**Table 4:** Number of patients whose response sentences were assigned to each of the 4 identified topics (n=98; a patient could mention >1 topic and the same topic in multiple sentences).

Topic	Patients mentioning topic, n (%)
Treatment pathway	49 (50)
Video content	43 (44)
AI avatar	17 (17)
No content	12 (12)

### Sentiment Distribution by Topic

To assess whether patients spoke about each topic positively or negatively, a grouped bar chart was created to show the sentiment of each response sentence and the topic with which it was associated. As shown in Figure 4, sentiment for the treatment pathway topic was mostly positive or neutral, and sentiment for the video content topic was mostly positive, whereas sentiment for the AI avatar topic was predominantly negative. This likely reflects that most patients (10/17, 59%) who mentioned the AI presenter described it as “impersonal,” which was the second most relevant word in sentences assigned to this topic.

### Word Clouds

To better understand the patients’ feedback about each aspect of the video and their associated feelings, word clouds were created to represent key words from the responses for each topic (Multimedia Appendix 1).

As shown in Multimedia Appendix 1, for the treatment pathway topic, it can be interpreted that patients found that they were reassured and felt calm, the treatment pathway was faultless, and many of them were thankful.

For the video content topic, positive words can be seen that likely describe how patients felt about the content, such as “informative,” “relevant,” “clear,” and “comprehensive.”

Finally, for the AI avatar topic, it can be interpreted that patients who mentioned the avatar felt that it was “impersonal” and felt “disconnected” from the topic of breast cancer treatment, or they found it “distract[ing].”

### Summarization

To identify the main takeaways from the patients’ feedback, the T5 abstractive summarization model was used to summarize response sentences from each topic (Textbox 1). The generated summaries were then manually edited and checked against the raw data for grammatical clarity and to confirm that they accurately represented the data. The summaries aligned with findings from the earlier analyses.

**Manually edited artificial intelligence (AI)–generated summaries of patient feedback by the text-to-text transfer transformer model by topic.**

**Treatment pathway**

- Many patients were pleased with the treatment process and felt that they were well informed throughout. The video was helpful, reassuring, and well explained. A few patients commented that they wished that they had this video at the start of the treatment.

**Video content**

- Most respondents were positive about the video content and found it to be informative, clear, concise, and easy to understand and felt that it covered the essential points of the follow-up program.

**AI avatar**

- Most respondents who mentioned the AI-generated presenter felt that the use of the presenter made the video feel impersonal and would have preferred a human presenter. A few respondents also found the AI presenter distracting, and a few mentioned that the voice was not in sync with the presenter’s lip movements.

## Discussion

### Principal Findings

This study evidences that patient health care education material delivered using GenAI (via an avatar) is generally received positively by patients. Moreover, algorithmic approaches such as NLP can be effectively applied to qualitative feedback to identify areas for improvement in delivering health information using this technology. This point is especially pertinent, given the current workforce constraints within an evolving NHS [66], which make it infeasible to manually analyze even a small corpus (n=98) of patient feedback.

In addition, this work reinforces earlier findings that large volumes of patient comments can be efficiently processed using NLP methodologies to draw meaningful and actionable insights [9,29,31,32,36-38,40,41,67]. This adds to the body of evidence demonstrating that various NLP techniques, such as topic modeling and sentiment analysis, and visualization of their results provide a more profound understanding of patient feedback, in this case revealing both successful aspects of the video and areas needing improvement. New visualization techniques were explored, such as sentiment-based color-coded word clouds by topic, which provided clearer insights into the data. In addition, as the NHS elective care recovery delivery plan [68] commits to providing better information and support to patients with the introduction of new and innovative ways of delivering health care, it is more important than ever that patients feel confident that their feedback is sought and integrated into new models of care. Consistent with the NHS plan’s objectives, this study demonstrated how summarization models can facilitate an overview of the patient data, significantly reducing the time required to review individual comments.

Overall, the analysis revealed predominantly positive responses. Four key topics were identified, with positive feedback on the treatment pathway and video content and negative feedback concerning the digital avatar. Many patients expressed satisfaction with their experience of the breast cancer treatment pathway, noting that the videos were informative and could be a valuable and reassuring resource for patients at the beginning of the treatment pathway. Concerns regarding the avatar included its robotic or impersonal voice and the audio being out of sync with the avatar’s lip movements. However, as this technology is ever improving and producing more human-like avatars, these concerns are likely to be resolved with further model development. Other patients did not like the use of AI to create these videos at all, which could be reflective of the general mistrust of AI in health care [69].

This underscores an important ethical consideration in the use of such technology for health care education. While our study demonstrates the value of using AI to quickly and effectively create valuable breast cancer educational videos, this technology may not be appropriate for all types of health care–related content. Specifically, AI-generated videos may not be suitable for discussing particularly sensitive or emotional topics. Furthermore, adherence to responsible AI regulation and guidelines, as well as transparent communication with patients about the role of AI in creating educational materials, is crucial to maintaining patient trust [70]. This is especially true considering the rise in misuse of GenAI technology to create videos to deceive people or spread misinformation [71-75]. Thus, it will be an important consideration when creating and distributing AI-generated educational videos to find ways to distinguish them as credible and reliable sources of information.

These results echo those of similar studies indicating that closed-ended questions do not disclose the full picture of patient feedback and that open-ended responses are crucial for understanding the nuances of patient opinions. Consistent with the findings of Gallan et al [28] in a similar study, this is further supported by the differences in sentiment between the patients’ overall video scores and their free-text responses. As shown in Figure 2, some patients assigned a perfect score yet left a negative or neutral comment. Although some of these negative or neutral comments were unrelated to the video itself, a negative comment regarding the respondent’s dislike of the avatar wearing black provides an actionable insight for improving these videos.

Our findings add to a wider discussion on the optimal methods for delivering health care information and promoting health literacy. Specifically, a large body of work has demonstrated that written health information is often difficult for most intended audiences to comprehend [76]. A systematic review of readability assessment studies reported that most of the COVID-19 vaccine information supplied by health care providers exceeded the recommended grade reading level, which the authors hypothesize contributed to vaccine hesitancy during the pandemic [77]. Another systematic review reported that web-based patient information for common sports injuries was written above the recommended reading level [78]. More recently, a systematic review of written information for patients spanning 30 years highlighted that the reading level of patient information across all clinical areas was too high for patients and encouraged new modes of communication to educate patients [79]. Given that almost half of the patients (48/98, 49%) in this study expressed positive sentiment toward health care information delivered using GenAI (in the form of an avatar), the reported high readability barriers may be considerably lowered by adopting this technological approach. Moreover, GenAI avatars can now automatically deliver content in >140 languages with 1-click translations [80], potentially further addressing language barriers in health care education [4].

Beyond providing effective patient education, these videos aim to help reduce the time that specialist breast cancer care nurses spend answering common patient queries, allowing them to focus on patient care and on answering more highly specialized, treatment-related questions. While data were not collected on the impact of these AI-generated videos on reducing health care professional workloads in this study, patient educational videos have been shown to reduce the number of patients requiring direct contact with health care providers [81]. Furthermore, previous studies have found that patients prefer this form of media over traditional leaflets and that educational videos improve shared decision-making in clinical practice while increasing health literacy and medication adherence [82,83]. However, further work is needed to confirm such beneficial impacts in our case, such as a comparison of the number of calls made by patients to breast cancer nurses before and after the introduction of these videos.

### Limitations

The primary limitations of this study were the small sample size (98 responses) and patient acuity. Although splitting the data into sentences increased the dataset size and provided deeper insights, future projects would benefit from a larger sample for more accurate NLP model evaluation and pattern investigation. More data would enable the labeling and testing of the topic and sentiment models, which would provide more robust and objective accuracy metrics. Furthermore, semantic analysis could improve by incorporating higher n-grams and refining contextual polarity [40,84]. Finally, larger sample sizes would likely allow for more effective summarization by topic and sentiment, potentially revealing deeper insights into patient responses.

If additional data cannot be collected, synthetic data generation [85] and augmentation techniques such as word replacement or shuffling could be used to enhance the dataset [86]; for example, Li et al [87] used the synthetic minority oversampling technique (SMOTE) algorithm to balance their dataset of patient complaints to ensure equal representation across topic categories.

Alternatively, improving the response rate for the open-ended question—38.4% (98/255) in this study—would help increase the sample size. This might be achieved by making the question mandatory, reordering the survey questions, or offering incentives to respondents who complete the full survey [88,89]. In this study, all survey questions were optional; however, in contrast to the low response rate for the open-ended questions, the closed-ended questions had a minimum response rate of 98.8% (252/255), with 9 (75%) of the 12 questions being answered by all respondents. This is expected because closed-ended questions are easier and faster to answer; however, future studies should explore ways to increase the response rate for open-ended questions to levels similar to those for closed-ended questions for a more diverse set of responses.

It is worth noting that there are limitations to our sentiment analysis methods. First, although the pretrained sentiment analysis model we used is known to generally perform at a high degree of reliability, not all sentiment categorizations made by the model will be completely accurate. Precise accuracy metrics specific to our model are unavailable. However, previous research studies indicate that similar models, based on the RoBERTa architecture, demonstrate accuracies ranging from 90% to 96% [63,90].

Second, it is important to understand that the color of each keyword in the word cloud is associated with the sentence in which the word appears, rather than the sentiment associated with the word itself. This is because we are using sentences as a way to segment the responses into constituent topics. Although this technique helps us better understand the details of patient responses, this is not always the case in reality; for example, a sentence could contain positive sentiment about one topic and negative sentiment about another. An improvement to this method would be to segment the responses by constituent topics, rather than relying on sentence boundaries, as in aspect-based sentiment analysis [91]; however, implementing this technique poses challenges and requires the adoption of more complicated models.

Only relevant data were collected to answer the primary research question in accordance with the UK government’s Caldicott Principles [92]. This was a necessary step in data governance and ethics approval; however, it limits the generalizability of our findings to diverse populations [93] because we did not have access to any demographic data. Factors including patient age, digital literacy, opinion on technology, patient acuity (eg, patient activation scores [94]), and patient confidence in managing their disease may all influence survey results [95]. Further analysis of the impact of these factors on the patient perspective on the use of these videos as patient education tools would help reveal whether the findings of this study represent those of broader and more diverse patient groups.

The patients with breast cancer included in this study were in remission; therefore, it is unknown whether patients with breast cancer who are undergoing active treatment regimens would have similar perspectives. This is an important consideration because the information in the 7 videos created using GenAI technology by this collaborative work is aimed at patients at different points in the treatment pathway and on different treatment regimens (eg, “Breast Pain after Surgery” for patients who have undergone mastectomy or “Endocrine Therapy” for patients receiving hormonal treatment). Furthermore, cancer stage may influence patients’ views of these videos because individuals at different stages may face varying health and social care challenges (eg, loss of a job or reduced working hours and childcare responsibilities).

Digital inclusivity is another important factor to consider because 96% of breast cancers are diagnosed in women aged >40 years [96], as a function of lower digital literacy and internet use [97,98]. Thus, it is crucial that the information in these videos is delivered in an accessible way to ensure that there is no disparity in the level of service provided to a hospital’s diverse patient population. Therefore, further investigation is warranted with a broader sample of patients with breast cancer to explore diverse prospective patient feedback throughout cancer treatments.

Finally, the survey response rate was 63.8% (255/400), with only 38.4% (98/255) of the respondents providing open-ended feedback. This low sample size for the open-ended free-text question may not capture the full spectrum of patient feedback. Moreover, there may exist a bias in the subset of patients with breast cancer who answered the free-text question and among those who completed the survey compared to the broader population of patients with breast cancer who completed the survey and those who were sent the survey, respectively (ie, nonresponse bias); for example, individuals with strong positive or negative opinions may be more likely to volunteer open-ended feedback, skewing the data [27]. This may have contributed to the predominance of negative comments concerning the digital avatar: it is possible that those who did not have strong opinions on the avatar did not provide free-text feedback; however, further research is required to confirm this.

Alternatively, factors such as digital literacy or age, as discussed previously, may have influenced a patient’s likelihood of providing feedback (at all or specifically open-ended) [88,99]. However, due to ethical and data privacy constraints, we cannot disclose any information about the patients who did not complete the study. This lack of comparative demographic data restricts our ability to understand and reveal any potential biases in the collected responses.

### Comparison to Prior Work

Unlike studies that analyzed unsolicited patient comments from social media or hospital feedback sites, this project used solicited survey data. Greaves et al [40] note that unsolicited comments often exhibit selection bias toward more extreme positive or negative experiences, resulting in unrepresentative, polarized results. By collecting feedback through surveys, this study minimized such bias, as evidenced by the high level of neutral feedback shown in Table 2.

Splitting responses into sentences enhanced granularity, improving the understanding of topics and sentiments, as shown by the differences in the proportions of negative, neutral, and positive sentences compared to full responses (Table 2). Nawab et al [41] also used sentence-level analysis but only when sentiments differed between sentences, which may have led to the omission of some topics. This approach also increased the dataset size from 98 to 167, benefiting NLP techniques that require larger datasets (namely, topic modeling, summarization, and TF-IDF word clouds). However, splitting by sentence can fail to capture all topics and sentiments accurately because sentences can contain both positive and negative sentiments. Consequently, the topic-based sentiment analysis may not be accurately representative in all cases, and word cloud colors may reflect sentence sentiment rather than the word’s overall sentiment. Splitting responses by constituent topics (multilabeling) could improve accuracy, but this approach is complex and may not suit short texts, such as those in this study (mean length 15 words, SD 10) [100,101].

The sentiment analysis results support prior research indicating that this technique can accurately assess patient survey responses [28,40,67]. However, due to the small sample size (n=167 sentences) and the absence of labeled data, a quantitative evaluation of model accuracy was not performed; instead, visual inspection was used to identify the most suitable model.

Similar to previous studies, simpler models such as VADER struggled with health-related comments [102]. As mentioned previously, this is likely because the negative scores of words such as “cancer” have a big impact on the overall sentence scores, given that VADER relies on a sentiment dictionary rather than taking into account the full context of the sentence. By contrast, BERT-based models, which have not been extensively explored for patient experience data, showed promise. This study is the first to apply the HuggingFace RoBERTa model [48] to patient data, although BERT-based models have been used previously; for instance, Osváth et al [103] reported 72% accuracy using fine-tuned huBERT on a Hungarian Twitter (subsequently rebranded as X) dataset, while Chatzimina et al [104] achieved accuracies of 95% and 91% with BERT and RoBERTa, respectively, fine-tuned on Greek patient-clinician conversations. In addition, RoBERTa-based models (trained on Wikipedia and news articles) fine-tuned on nonpatient data have demonstrated accuracies ranging between 90% and 96% [63,90].

In line with previous studies [32,36,105], unsupervised topic modeling effectively identified unknown topics in responses. BERTopic with KeyBERT for keyword extraction and k-means clustering emerged as the best model. This was assessed through both topic coherence (a quantitative metric) and a subjective evaluation of topic relevance. Most studies rely solely on topic coherence to evaluate models [106], but in support of the evaluation methods used in this work, previous studies [107-109] argue for taking topic usefulness into account, with the addition of incorporating human-interpretability metrics such as word intrusion and topic intrusion. These techniques involve human assessors identifying “intruder” words or topics, helping to mitigate bias and better gauge the model’s usefulness. Future studies should include these methods with multiple assessors for a more comprehensive evaluation.

Consistent with the findings of Egger and Yu [110], BERTopic performed better with shorter, emotional texts than LDA and NMF. This is attributed to BERT embeddings, which capture contextual information and can effectively process diverse text elements such as emojis and punctuation. By contrast, LDA and NMF rely on a bag-of-words approach, which ignores word order and context, making them more sensitive to noise and nonstandard text elements. Furthermore, Steele et al [111] similarly found success in applying BERTopic to identify care experience topics in a much larger dataset of approximately 47,000 free-text responses from patient experience surveys. The findings support the use of this method for revealing topics in unstructured patient survey response data in future, larger studies.

In this study, k-means clustering was used to assign every sentence to a topic, ensuring no data loss and the inclusion of all patient responses in the analysis. However, this method may have compromised topic quality by oversimplifying data and adding noise, as indicated by the lower topic coherence score when switching from hierarchical density-based spatial clustering of applications with noise (HDBSCAN) to k-means clustering (Table 1). Nevertheless, combining k-means clustering with KeyBERT achieved the highest topic coherence.

An interesting finding from the study was the negative color-coded occurrence of the term “GP” in the treatment pathway word cloud (Multimedia Appendix 1). This is particularly noteworthy, considering this study’s cohort: patients who had been through the treatment pathway and, therefore, should have had no contact with primary care providers such as general practitioners (GPs). Initially, it was thought that the negative color coding of this term might reflect the recent decline in patient satisfaction with GP access [112,113]. However, further investigation revealed that the contexts in which “GP” appeared were neutral (despite the sentences concerned being correctly classified as negative overall). Specifically, patients referred to GPs because, prior to watching the educational video, they were unaware that they should recontact their secondary care provider (breast cancer nurses) with any queries, rather than their GPs. This observation ties into broader issues surrounding cancer treatment pathways within the NHS.

The COVID-19 pandemic has exacerbated backlogs in cancer care [114,115]. For patients with a history of cancer, clear guidance on whom to contact in the event of a suspected recurrence is critical because such clarity is linked to improved health outcomes [116]. As highlighted by Breast Cancer Now, the lack of clarity can result in patients seeking help from GPs [116], and this pattern is reflected in the findings of this study. These results underscore the importance of these educational videos in supporting patients to contact the right service at the right time.

Abstractive summarization models were evaluated based on the quality and readability of their outputs. Recall-Oriented Understudy for Gisting Evaluation (ROUGE) *F*-measure scores [117] are a standard metric for evaluating summarization performance. They measure the n-gram lexical overlap between the generated summary and the reference human-written “gold” summary [118]. Although a gold summary did not exist to use as a benchmark in this project, an improvement in future similar studies would be to identify a validation dataset to quantitatively assess different summarization models using this metric, such as a previous patient survey and a human-written summary of the results.

Through manual inspection, the T5 model performed best in summarizing patient feedback responses; the summaries generated by Pegasus, FLAN-T5, bidirectional and auto-regressive transformers, and GPT-2 were not as clear or comprehensive. This supports the findings of Nguyen et al [119], who reported in their comparative evaluation of many popular summarization models that T5-generated summaries were the most readable and that Pegasus performed relatively poorly. Similarly, Tariq et al [44] found success using the T5 model to summarize health-related texts. In this study, GPT-2 did not generate comprehensive summaries. However, Fu et al [120] report that GPT-2 performs better when given ≥1 examples (few-shot learning) and that GPT-3 performs even better; therefore, these avenues should be explored in future related work. Interestingly, FLAN-T5—the instruction-tuned successor to T5—performed worse than the original T5 model in this study. This contrasts with the findings of Pal et al [121], who reported superior ROUGE scores for FLAN-T5 in generating hospital discharge summaries. However, this is likely due to the differences in evaluation methods: Pal et al [121] used ROUGE scores to benchmark generated summaries against “ground truth” discharge summaries, whereas this study relied on human-based preference evaluation due to the absence of reference “gold” summaries.

### Conclusions

This work demonstrates the utility of GenAI videos for delivering patient information and the success of NLP techniques in accurately and efficiently generating insights into patient feedback. Sentiment analysis showed that 81% (79/98) of the patient responses were positive or neutral, with negative comments mostly related to the AI avatar. Using the BERTopic model, we identified 4 topics: the breast cancer treatment pathway, video content, the digital avatar, and short responses with little or no content. The T5 model was used to generate abstractive summaries of the responses by topic. Combining the results from these NLP techniques facilitated the creation of clear visuals and insights, including the novel combination of topic and sentiment analysis with TF-IDF to produce sentiment-based color-coded word clouds by topic. These deeper analyses revealed valuable insights into the patients’ perspectives on the video, with many commenting that they found the video informative and reassuring and that they believed that it would be a valuable resource for individuals beginning the breast cancer treatment pathway. The positive results from this work validate the use of GenAI in creating patient educational videos. Negative patient feedback mostly concerned technical issues related to the avatar that are likely to be resolved with further model development; however, similar future investigations into patient opinions will be needed to confirm this.

To assess how well such GenAI videos meet the varied needs and preferences of the wider patient population, future studies should aim to recruit a larger and more diverse sample of patients with breast cancer, spanning different ages, digital literacy levels, geographies, stages of treatment, treatment regimens, and levels of patient acuity. In combination with an improvement in the free-text question response rate, this would also help to mitigate any potential impact of bias on the collected responses. Given patient consent and compliance with data privacy regulations, collecting demographic data such as age and literacy levels alongside response data would also allow for the investigation of potential biases in patient feedback, thereby improving the usability of study findings. A larger dataset would also enable more robust evaluations of NLP models, especially when combined with expert annotations of sentiment and topics as well as ground truth summaries.

Overall, this study provides insight into the potential of GenAI to create patient educational videos, while identifying patient concerns that can be addressed in future AI-generated digital materials. The NLP methods applied here can be used in future, larger studies to automate efficient analysis and insight generation from unstructured patient response data.

## Appendix

Multimedia Appendix 1 Word clouds representing keywords extracted from patient responses for each topic. Keywords are color coded according to the sentiment of the sentences in which they appeared. For words appearing in many sentences, sentiment scores were aggregated, and the sentiment with the highest probability determined the overall sentiment context for that word. Colors indicate sentiment as follows: red for negative, orange for neutral, and green for positive.

## Abbreviations

AI: artificial intelligence
BERT: bidirectional encoder representations from transformers
FLAN: fine-tuned language net
GenAI: generative artificial intelligence
GP: general practitioner
HDBSCAN: hierarchical density-based spatial clustering of applications with noise
HUTH: Hull University Teaching Hospitals NHS Trust
LDA: latent Dirichlet allocation
LLM: large language model
NHS: National Health Service
NLP: natural language processing
NMF: nonnegative matrix factorization
RoBERTa: Robustly Optimized BERT Pretraining Approach
ROUGE: Recall-Oriented Understudy for Gisting Evaluation
T5: text-to-text transfer transformer
TF-IDF: term frequency–inverse document frequency
VADER: Valence Aware Dictionary and Sentiment Reasoner

## Data Availability

The datasets generated or analyzed during this study are not publicly available due to patient data protection regulations but are available from the corresponding author on reasonable request.
