# Supplementary figures and images for: Using Natural Language Processing to Explore Patient Perspectives on AI Avatars in Support Materials for Patients With Breast Cancer: Survey Study
Source: J Med Internet Res. 2025 Jun 20;27:e70971. doi: 10.2196/70971 (PMC12228011; doi:10.2196/70971)

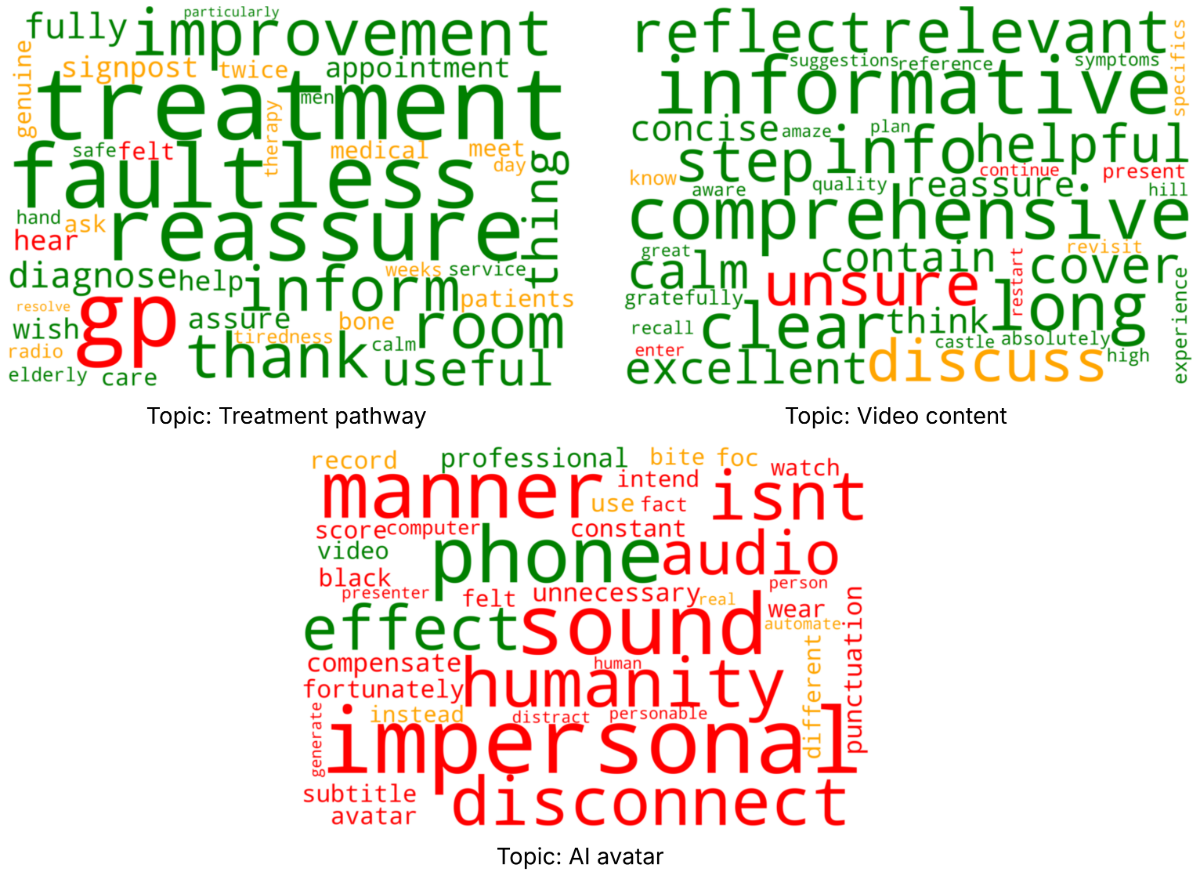

Supplement: Multimedia Appendix 1 [file jmir_v27i1e70971_app1.png]
